# A Series of New Ligustrazine-Triterpenes Derivatives as Anti-Tumor Agents: Design, Synthesis, and Biological Evaluation

**DOI:** 10.3390/ijms160921035

**Published:** 2015-09-02

**Authors:** Bing Xu, Fuhao Chu, Yuzhong Zhang, Xiaobo Wang, Qiang Li, Wei Liu, Xin Xu, Yanyi Xing, Jing Chen, Penglong Wang, Haimin Lei

**Affiliations:** 1 School of Chinese Pharmacy, Beijing University of Chinese Medicine, Beijing 100102, China; E-Mails: weichenxubing@126.com (B.X.); chufhao@163.com (F.C.); lq_cn@126.com (Q.L.); xu.xin@vip.126.com (X.X.); xingyanyi1988@126.com (Y.X.); cjttmc@sina.cn (J.C.); 2 Department of Pathology, Beijing University of Chinese Medicine, Beijing 100102, China; E-Mail: zyz100102@126.com; 3 Center of Scientific Experiment, Beijing University of Chinese Medicine, Beijing 100102, China; E-Mail: xiaobo1001@gmail.com; 4 School of Management, Beijing University of Chinese Medicine, Beijing 100102, China; E-Mail: nolanlucky@163.com

**Keywords:** ligustrazine-triterpenes derivatives, cytotoxicity selectivity, combination principles, apoptosis

## Abstract

A series of novel ligustrazine-triterpenes derivatives was designed, synthesized and screened for their cytotoxicity against five cancer cell lines (Bel-7402, HepG2, HT-29, Hela, and MCF-7) and Madin-Darby canine kidney (MDCK). Current study suggested that most of the ligustrazine-triterpenes conjunctions showed better cytotoxicity than the starting materials. In particular, compound **4a** exhibited better cytotoxic activity (IC_50_ < 5.23 μM) against Bel-7402, HT-29, MCF-7, Hela, and HepG2 than the standard anticancer drug cisplatin (DDP). The cytotoxicity selectivity detection revealed that **4a** exhibited low cytotoxicity (IC_50_ > 20 μM) towards MDCK cells. A combination of fluorescence staining observation and flow cytometric analysis indicated that **4a** could induce HepG2 cell apoptosis. Further studies suggested that **4a**-induced apoptosis is mediated through depolarization of the mitochondrial membrane potential and increase of intracellular free Ca^2+^ concentration. In addition, the structure-activity relationships of these derivatives were briefly discussed.

## 1. Introduction

Natural products play a highly significant role in the development process of drug discovery [1]. This was particularly evident in the areas of anti-cancer drugs; over 60% of FDA approved anti-tumor drugs were shown to be of natural origin, such as the best known anticancer drugs vinblastine, etoposide and paclitaxel [2]. In recent years, several pentacyclic triterpenoids, such as oleanolic acid (OA), ursolic acid (UA), glycyrrhetic acid (GA) and betulinic acid (BA), were found to possess remarkable anti-proliferative and apoptosis-inducing activity in various cancer cell lines [3,4,5,6]. Moreover, pentacyclic triterpenoids and their derivatives possessed strong selective cytotoxicity on human carcinoma cells, but not on normal cells [7,8,9,10]. This has stimulated interest in using pentacyclic triterpenoids as the scaffold to synthesize new anticancer agents [11,12,13,14,15,16].

Structural modification of bioactive natural products according to combination principle is an important approach in the search for new lead compounds [17,18,19,20]. In our previous work of this field, we had designed and synthesized a series of ligustrazine-triterpenes derivatives, among which some compounds showed promising selective cytotoxicity on human carcinoma cells. It revealed that the introduction of ligustrazine could increase the cytotoxicity and selectivity of these compounds [17,20]. Moreover, the oral bioavailability of ligustrazine-triterpenes ester derivative was checked using a rat model. Ligustrazine-triterpenes ester derivative was metabolic stability following oral administration [21,22].

In our continuous effort to develop new antitumor agents, we designed and synthesized a series of novel ligustrazine derivatives by combination ligustrazine with several pentacyclic triterpenoids. All newly synthesized compounds were fully characterized. With their cytotoxicity was evaluated on a panel of human tumor cell lines, including Bel-7402, HepG2, HT-29, Hela, MCF-7. The antineoplastic drug cisplatin (DDP) was selected as the positive control, with the Madin-Darby canine kidney cell line (MDCK) was used to test the nephrotoxicity of the ligustrazine-triterpenes derivatives and DDP. The antitumor mechanism was also investigated by fluorescence staining observation and flow cytometric analysis on treated HepG2 cells. In addition, the structure-activity relationships (SARs) of these novel compounds were also discussed.

## 2. Results and Discussion

### 2.1. Chemistry

**Scheme 1:**
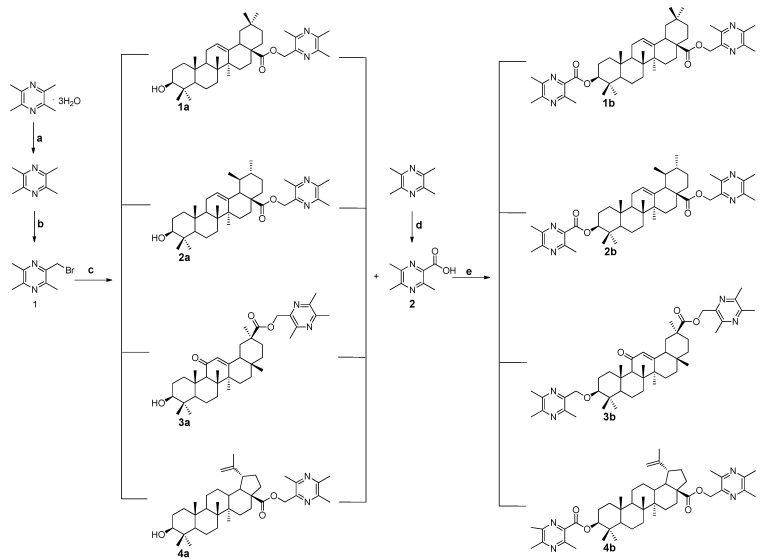
Synthesis of the ligustrazine-triterpenes derivatives **1a**–**4a**, **1b**–**4b**. Reagents and Conditions: (**a**) Benzene, reflux, 10 h; (**b**) CCl_4_, *N*-bromobutanimide (NBS), *hv*, reflux, 12 h; (**c**) *N*,*N*-Dimethylformamide (DMF), K_2_CO_3_, N_2_, 85 °C, 1.5 h; (**d**) H_2_O, KMnO_4_, 37 °C, 6 h; and (**e**) dry CH_2_Cl_2_, EDCI, DMAP, 12 h.

**Scheme 2:**
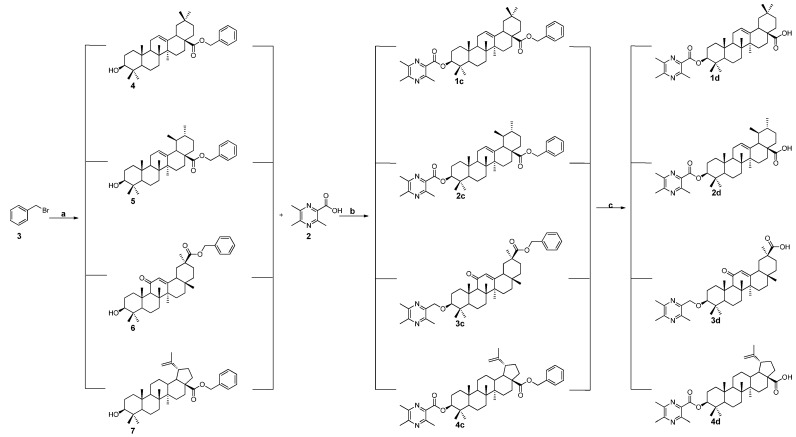
Synthesis of the ligustrazine-triterpenes derivatives **1c**−**4c**, **1d**−**4d**. Reagents and Conditions: (**a**) DMF, K_2_CO_3_, N_2_, 85 °C, 1.5 h; (**b**) dry CH_2_Cl_2_, EDCI, DMAP, 12 h; and (**c**) Methanol (MeOH), 10% Pd(OH)_2_/C, H_2_, 12 h.

All the designed derivatives were synthesized via the routes outlined in Scheme 1 and Scheme 2. As the important intermediates, 2-(bromomethyl)-3,5,6-trimethylpyrazine (**1**) and 3,5,6-trimethylpyrazine-2-carboxylic acid (**2**) were prepared according to our previous study [17]. As shown in Scheme 1, the pentacyclic triterpenes underwent alkylation reaction with the intermediate **1** to afford compounds **1a**–**4a**. Subsequently, Compounds **1a**–**4a** were further reacted with **2** in the presence of 1-(3-dimethylaminopropyl)-3-ethylcarbodiimide hydrochloride (EDCI) and 4-dimethylaminopyridine (DMAP) to generate the target compounds **1b**–**4b**.

The synthesis of compounds **1c**–**4c** and **1d**–**4d** were conducted according to the procedures in Scheme 2. First, the pentacyclic triterpenes were reacted with benzyl bromide under alkaline conditions to form the intermediates **4**, **5**, **6** and **7**. Subsequently, the intermediates were further treated with **2** to generate the target compounds **1c**–**4c**, respectively. Finally, the benzyl groups of compounds **1c**–**4c** were taken off by catalytic hydrogenolysis over Pd(OH)_2_/C to give the target compounds **1d**–**4d**. The structures of all target compounds were confirmed using ^1^H-NMR, ^13^C-NMR and high-resolution mass spectrometer (HRMS).

### 2.2. Cytotoxicity Assay

The cytotoxicity of ligustrazine-triterpenes derivatives was evaluated on five tumor cell lines (Bel-7402, HepG2, HT-29, Hela, and MCF-7) using the standard MTT assay. In the study, the antineoplastic drug cisplatin (DDP) was selected as the positive control. The MDCK cell line was used to test the toxicity of the ligustrazine-triterpenes derivatives and DDP. IC_50_ values for different cell lines are reported in Table 1. After combination with ligustrazine, most of the synthesized compounds showed significantly improved cytotoxicity compared to the starting materials. Among the candidates, compounds **1a**, **1d**, **4a** and **4d** exhibited much better antiproliferative activities than the starting materials on all tested cell lines. The cytotoxicity selectivity detection revealed that most of the ligustrazine-triterpenes derivatives exhibited low cytotoxicity towards MDCK cell. In particular, compound **4a** exhibited better cytotoxic activity (IC_50_ < 5.23 μM) against Bel-7402, HT-29, MCF-7, Hela and HepG2 than the standard anticancer drug DDP, while it showed lower cytotoxicity (IC_50_ > 20 μM) than DDP (IC_50_ = 14.61 μM) on MDCK cells.

From the obtained results, it was observed that the derivatives containing single-ligustrazine substituents exhibited better antiproliferative activities than those containing bis-ligustrazine substituents, as exemplified by **1a** > **1b**, **2a** > **2b**, **3a** > **3b**, **4a** > **4b**. Structure-activity relationship analysis among **1a**–**1d**, **2a**–**2d** also revealed that the compounds with oleanane skeleton seemed to be more active than those with ursane skeleton, such as **1a** > **2a**, **1b** > **2b**, **1c** > **2c**, **1d** > **2d**, which was in agreement with the previously published study [23]. In addition, it was observed the derivatives containing single-ligustrazine substituents seemed to be more active than those with benzyl substituent at C-28 or C-30, such as **1d** > **1c**, **2d** > **2c**, **4d** > **4c**. These findings may provide a new framework for the design of new ligustrazine-triterpenes derivatives as anti-tumor drugs. Based on the above evidence, **4a** (IC_50_ < 5.23 μM) was selected for further analysis including its cytotoxicity selectivity and its mechanism of growth inhibition on HepG2 cell lines.

**Table 1:** Anti-proliferative effects and ClogP values of the target compounds and starting materials.

Compound	IC_50_ (μM)						ClogP
	Bel-7402	HT-29	HepG2	MCF-7	Hela	MDCK	
**TMP, OA, UA, GA, BA**	>20 ^a^	>20	>20	>20	>20	ND ^b^	NC ^c^
**1a**	10.24	13.22	14.85	15.17	14.07	>20	9.72
**1b**	>20	>20	12.09	10.55	>20	>20	11.26
**1c**	>20	>20	>20	>20	>20	ND	12.32
**1d**	9.28	8.43	7.94	5.69	4.37	16.39	10.16
**2a**	8.01	17.61	>20	>20	19.96	>20	9.72
**2b**	>20	>20	>20	>20	>20	ND	11.26
**2c**	>20	>20	>20	>20	>20	ND	12.32
**2d**	10.14	9.70	>20	9.49	9.66	>20	10.16
**3a**	>20	19.87	>20	>20	11.17	>20	7.57
**3b**	>20	>20	>20	>20	>20	>20	9.08
**3c**	>20	>20	9.06	7.58	17.51	>20	10.14
**3d**	>20	>20	>20	>20	>20	ND	7.99
**4a**	4.19	5.23	4.48	4.23	4.34	>20	9.57
**4b**	>20	>20	13.20	12.87	>20	>20	11.11
**4c**	>20	>20	>20	>20	>20	ND	12.17
**4d**	13.35	8.38	7.42	6.22	12.15	>20	10.01
**DDP**	5.91	5.28	4.57	5.17	6.22	14.61	NC

^a^ IC_50_ > 20.0 μM. We set a strict standard to the evaluation of antiproliferative activities. The maximal concentration of tested compounds is 20.0 μM. When IC_50_ > 20.0 μM, we considered the antiproliferative activities of the compounds were too weak to do further research; ^b^ “ND” not determined, due to the weak cytotoxicity, IC_50_ value of compounds on MDCK cells was not be detected; ^c^ “NC” means the ClogP value was not calculated.

### 2.3. Computer Simulation of ClogP

Optimization of solubility and hydrophobicity is very important in the drug discovery process because these properties are closely associated with absorption, distribution, metabolism, and excretion (ADME) properties of the compounds [24,25,26]. LogP, the logarithm of the octanol/water partition coefficient, is the most widely used measure of the lipophilicity [27]. In this work, the logP values for these derivatives were calculated using the Sybyl-X 2.0 software (Tripos, Certara Inc., St. Louis, MO, USA). As shown in Table 1, most of the derivatives with lower ClogP seemed to exhibited better anti-proliferative effects than those with higher ClogP, as exemplified by **1a**, **1d** > **1b**, **1c**; **2a**, **2d** > **2b**, **2c**; **4a**, **4d** > **4b**, **4c**. This was accordance with the previous study that triterpenes derivatives with lower Clogp display better cytotoxicity [12,28].

### 2.4. Preliminary Investigation of the Cytotoxicity Selectivity and the Apoptosis-Inducing Effect of **4a**

#### 2.4.1. Cytotoxicity Selectivity

**Figure 1:**
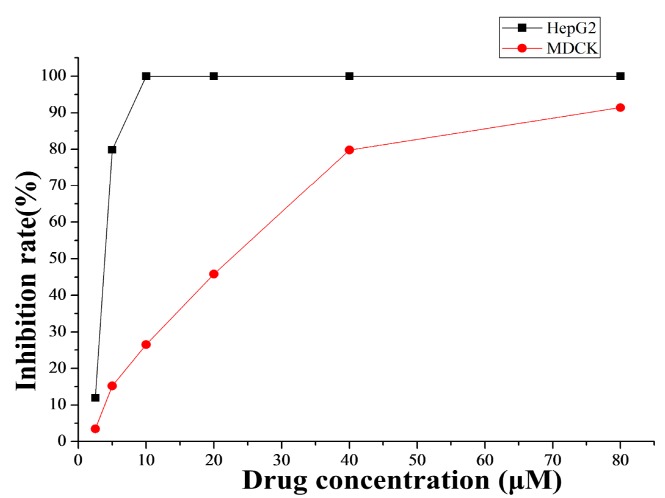
Cytotoxicity of **4a** against HepG2 tumor cells and MDCK cells. The cytotoxicity of **4a** was determined by MTT assay.

The treatment with increased doses of **4a** (Figure 1) by MTT assay showed that the inhibition rate against MDCK was just about 26.50% at the concentration of 10 μM, while the inhibition rate against human hepatoma cell HepG2 was up to 99.87% at the same concentration. Therefore, at least in part, compound **4a** had cytotoxicity selectively to human hepatocellular carcinoma cells *in vitro*.

#### 2.4.2. Morphological Observation of HepG2 Cells Using Giemsa Staining

**Figure 2:**
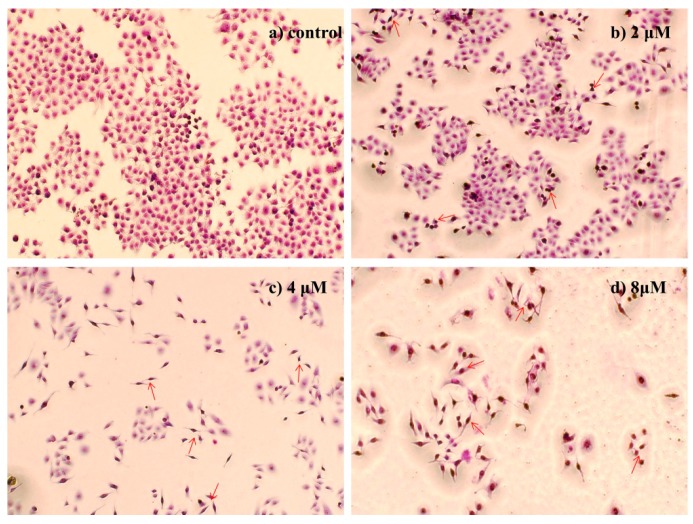
Morphological observation of HepG2 cells using Giemsa staining (200×): (**a**) Control group; (**b**) 2 μM; (**c**) 4 μM; and (**d**) 8 μM. The cell morphology was observed under the light microscope after Giemsa staining. The most representative fields are shown. Arrows indicate the typical apoptotic cell.

Morphological analysis of HepG2 cells using Giemsa staining showed that the cells in control group appeared normal morphology whereas **4a** treatment (2, 4, 8 μM for 72 h) caused evident nuclear fragmentation, chromatin condensation in a dose dependent manner (Figure 2), all of which were characteristic morphological alterations associated with apoptosis.

#### 2.4.3. Morphological Observation of HepG2 Cells Using DAPI Staining

**Figure 3:**
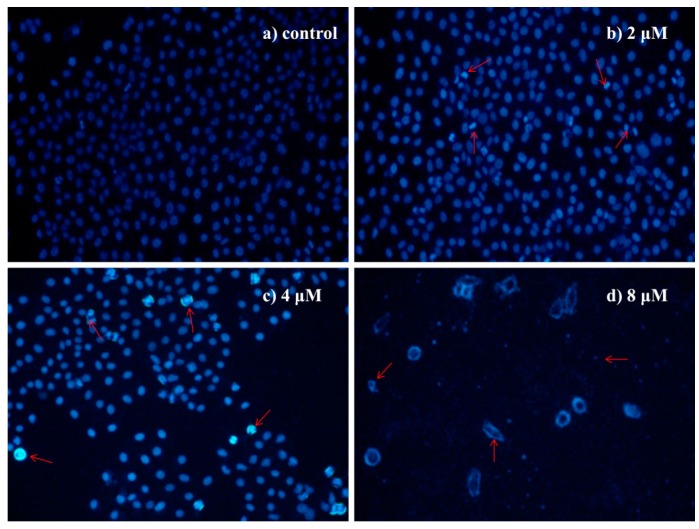
Morphological observation of HepG2 cells using DAPI staining (200×): (**a**) control group; (**b**) 2 μM; (**c**) 4 μM; and (**d**) 8 μM. The cell morphology was observed under the fluorescence microscope after DAPI staining. The most representative fields are shown. Arrows indicate the typical apoptotic cell.

DAPI staining further inferred that HepG2 cells treated with **4a** (2, 4, 8 μM) for 72 h displayed morphological features of apoptosis, such as nuclear condensation and fragmentation, the formation of apoptotic bodies with irregular shape, while untreated cells appeared normal (Figure 3).

#### 2.4.4. Morphological Observation of HepG2 Cells Using Acridine Orange/Ethidium Bromide (AO/EB) Double Staining

**Figure 4:**
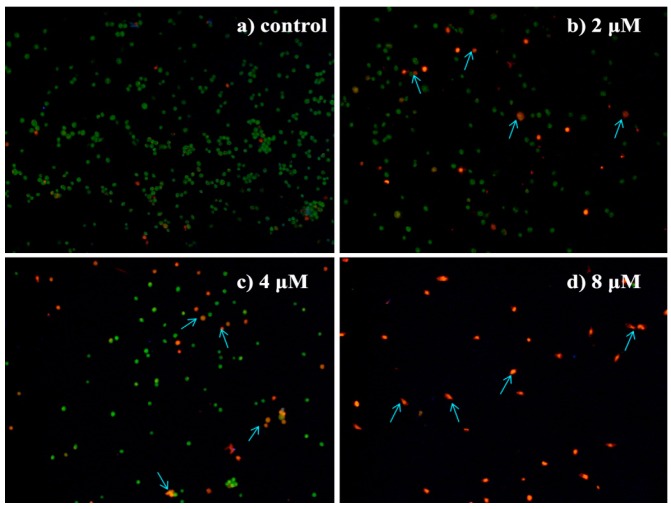
Morphological observation of HepG2 cells using AO/EB double staining (200×): (**a**) control group; (**b**) 2 μM; (**c**) 4 μM; and (**d**) 8 μM. The cell morphology was examined under the fluorescence microscope after AO/EB staining. The most representative fields are shown. Arrows indicate the typical apoptotic cell.

To further characterize **4a**-induced HepG2 cell apoptosis, the changes in cell morphology of the treated cells were determined using AO/EB double staining. AO is a vital dye that stains both live and dead cells as it can penetrate normal cell membrane. EB stains only cells that have lost their membrane integrity. Cells that stained green indicate viable cells, yellow indicate early apoptosis and orange/red indicate late apoptosis [29,30]. As shown in Figure 4, HepG2 cells in control group appeared bright green with normal cell morphology whereas the cells treated with **4a** (2, 4, 8 μM) for 72 h showed an increased number of orange- and red-stained cells in a dose dependent manner. And characteristic changes of apoptosis, including condensation and fragmentation of nucleus and formation of apoptotic bodies, were observed in the cells treated with different concentrations of compound **4a**. These results confirmed that **4a** significantly induced apoptosis in HepG2 cells.

#### 2.4.5. Apoptosis Analysis by Flow Cytometric Using Annexin V-FITC/Propidium Iodide (PI) Staining

**Figure 5:**
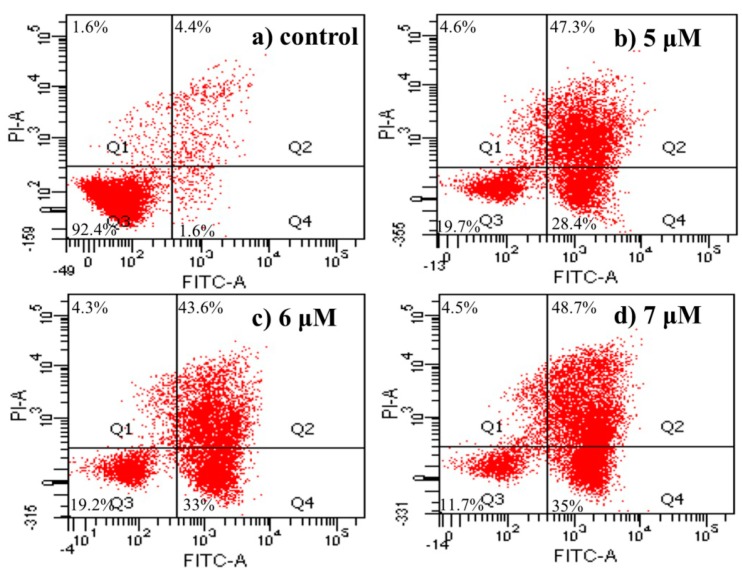
Apoptosis analysis by FCM using AnnexinV-FITC/PI staining: (**a**) control group; (**b**) 5 μM; (**c**) 6 μM; and (**d**) 7 μM.

To substantiate the ability of **4a** to induce apoptosis, further analysis was carried out using Annexin V-FITC/PI double staining technique in HepG2 cells. Cells stained with Annexin V-FITC and PI were classified as necrotic (Q1; Annexin^−^/PI^+^), late apoptotic (Q2; Annexin^+^/PI^+^), intact (Q3; Annexin^−^/PI^−^), or early apoptotic (Q4; Annexin^+^/PI^−^) cells. The results are shown in Figure 5. When HepG2 cells were treated with different concentrations of **4a** (5, 6, 7 μM) for 72 h, the apoptosis ratios (including the early and late apoptosis ratios) increased from 6% of the control to 75.7%, 76.6%, 83.7%, respectively. Even at the minimum concentration of 5 μM, **4a** induced cell apoptosis at a ratio of 75.7% after 72 h of treatment. Taken together, these results demonstrated that **4a** could induce HepG2 cells apoptosis in a concentration-dependent manner.

#### 2.4.6. Measurement of Mitochondrial Membrane Potential

**Figure 6:**
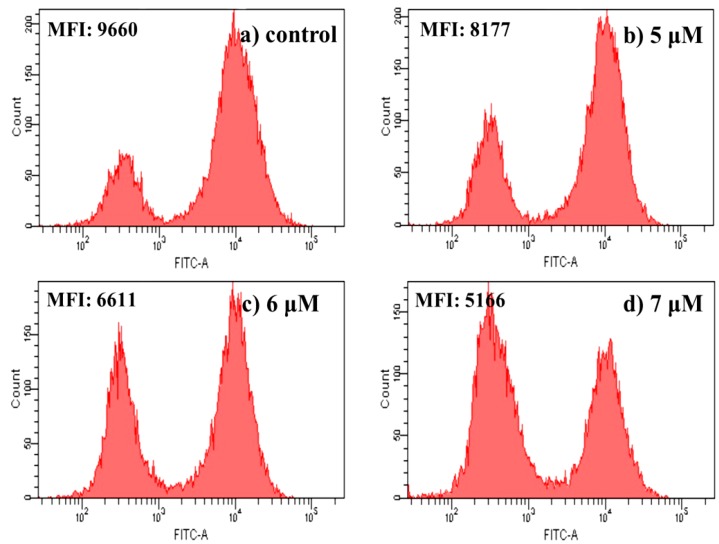
Effect of **4a** on mitochondrial membrane potential: (**a**) control group; (**b**) 5 μM; (**c**) 6 μM; and (**d**) 7 μM. Cells were determined by flow-cytometric analysis stained with Rhodamine 123 for 30 min. Results are expressed as mean fluorescent intensity (MFI).

Mitochondria play an essential role in the activation and progression of apoptosis. Mitochondrial membrane potential (*ΔΨ*m) is an important parameter of mitochondrial function. Depolarization of *ΔΨ*m is used as an early apoptotic maker in cells. In order to gain a better understand of the mechanism of **4a**-induced HepG2 cells apoptosis, depolarization of *ΔΨ*m was measured quantitatively by the fluorescent dye Rhodamine 123 (Rh123). As a cell-permeable cationic dye, Rh123 preferentially enters mitochondria based on the highly negative mitochondrial membrane potential. Depolarization of the membrane results in the loss of Rh123 from the mitochondria and a decrease in intracellular fluorescence intensity [31,32]. Flow cytometric analysis of HepG2 cells after treatment with different concentrations of **4a** (5 μM, 6 μM, 7 μM) for 72 h revealed that the fluorescent intensity decreased with the increase of **4a** concentration (Figure 6). That indicated **4a** was able to induce mitochondrial membrane potential disruption in HepG2 cells. This was in agreement with other reports that betulinic acid and its derivatives could induce depolarization of mitochondrial transmembrane potential [33,34].

#### 2.4.7. Intracellular Free Ca^2+^ Detection

**Figure 7:**
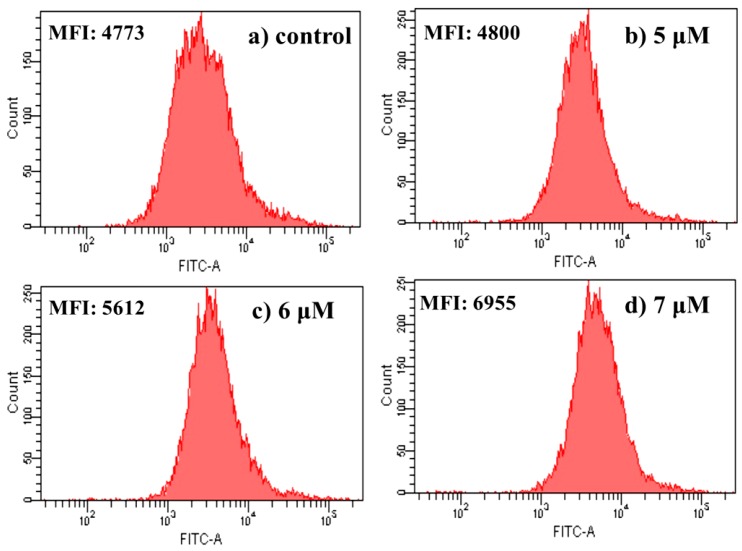
Effect of **4a** on intracellular free Ca^2+^ in HepG2 cells: (**a**) control group; (**b**) 5 μM; (**c**) 6 μM; and (**d**) 7 μM. After being treated with 5.0, 6.0 and 7.0 μM 4a for 72 h, cells were determined by flow-cytometric analysis stained with Fluo-3AM for 30 min. Results are expressed as mean fluorescent intensity (MFI).

A sustained increase in intracellular Ca^2+^ concentrations is recognized as a factor for cell death or injury [35]. The level of intracellular free Ca^2+^ is examined by the fluorescent dye Fluo-3AM. The Fluo-3 AM is converted to Fluo-3 upon deacetylation within the cells, and Fluo-3 increases green fluorescence upon Ca^2+^ binding [36]. As shown in Figure 7, with the increase of **4a** concentration, the level of intracellular Ca^2+^ increased steadily compared with the control group. As the concentration of **4a** increased from 5.0 to 6.0 to 7.0 μM, intracellular free Ca^2+^ fluorescence increased dramatically (from 4800 to 5612 to 6955). It was in accordance with the tendencies of mitochondrial membrane potential and cell apoptosis. The results indicated that the increase of intracellular Ca^2+^ was related with **4a**-induced HepG2 cell apoptosis.

## 3. Experimental Section

### 3.1. Chemistry

Reactions were monitored by TLC using silica gel coated aluminum sheets (Qingdao Haiyang Chemical Co., Qingdao, China) and visualized in UV light (254 nm). ^1^H-NMR and ^13^C-NMR assays were recorded on a BRUKER AVANCE 500 NMR spectrometer (Fällanden, Switzerland) with tetramethylsilane (TMS) as an internal standardm and chemical shifts are reported in δ (ppm). HR-MS were obtained by using Thermo Sientific TM LTQ Orbitrap XL hybrid FTMS instrument (Thermo Technologies, New York, NY, USA). Melting points (uncorrected) were measured at a rate of 5 °C/min using an X-5 micro melting point apparatus (Beijing, China). Cellular morphologies were observed using an inverted fluorescence microscope (Olympus IX71, Tokyo, Japan). Flash chromatography was performed using 200–300 mesh silica gel. The yields were calculated based on the last step reaction. All solvents and chemicals used were analytical or high-performance liquid chromatography grade.

*2-(Bromomethyl)-3,5,6-trimethylpyrazine* (**1**). Compound **1** was prepared according to our previously reported method [17]. The crude product, with 70% purity, was not purified further as it caused a strong mucous membrane irritation.

*3,5,6-Trimethylpyrazine-2-carboxylic acid* (**2**). Compound **2** was prepared according to the method described by Li *et al.*, with minor modifications [37]. To a solution of trimethylpyrazine (5.0 g, 36.8 mmoL) in water (200 mL), aqueous potassium permanganate (KMnO_4_) solution (8.6 g KMnO_4_: 150 mL water) was added dropwise at room temperature over about 60 min. Upon completion of the addition, the mixture was stirred at 50 °C for 12 h, then the warm reaction mixture was filtered and washed with hot water (300 mL, 90 °C). The filtrate and washing liquor were combined, cooled to 0–5 °C, and the pH adjusted to 2.0 with concentrated hydrochloric acid. Extraction was performed with ethyl acetate (200 mL × 3), and the organic phase was dried with anhydrous sodium sulfate. The solvent was removed by distillation under vacuum, and the residue was recrystallized from acetone to produce a light yellow solid (2.39 g, yield: 47.8%), m.p.: 162–163 °C.

#### 3.1.1. General Procedure for the Preparation of Ligustrazine-Triterpenes Derivatives **1a**–**4a**

Compound **1** (5.0 mmoL) and the corresponding pentacyclic triterpenes (5.0 mmoL) were dissolved in 25 mL dry DMF, then K_2_CO_3_ (5.0 mmoL) was added and the mixture was kept at 85 °C for 1.5 h under nitrogen atmosphere. The warm reaction mixture was poured into ice-water and the crude product was extracted with ethyl acetate. After drying the organic layer over anhydrous Na_2_SO_4_ and evaporating the solvent under vacuum, the crude product was purified by flash chromatography (Petroleum ether:acetone = 10:1).

*3β-Hydroxyolea-12-en-28-oic acid-3,5,6-trimethylpyrazin-2-methyl ester* (**1a**); *3-Hydroxy-11-oxoolean-12-en-30-oic acid-3,5,6-trimethylpyrazin-2-methyl ester* (**3a**). Both **1a** and **3a** were prepared according to our previous reported method [17].

*3β-Hydroxyurs-12-en-28-oic acid-3,5,6-trimethylpyrazin-2-methyl ester* (**2a**)*.* White solid, m.p.: 125.9–126.7 °C, yield 55.7%. ^1^H-NMR (CDCl_3_) (ppm): 0.60, 0.80, 0.91, 1.00, 1.01 (s, each, 3H, 5 × -CH_3_), 0.85 (d, *J* = 6.5 Hz, 3H, -CH_3_), 0.95 (d, *J* = 6.5 Hz, 3H, -CH_3_), 2.26 (d, *J* = 11.5 Hz, 1H), 2.51 (s, 3H, -CH_3_), 2.53 (s, 3H, -CH_3_), 2.58 (s, 3H, -CH_3_), 3.23 (m, 1H), 5.20 (brs, 1H, =CH-), 5.25, 5.06 (each, d, *J* = 12.0 Hz, 1H, -CH_2_), 1.00–2.50 (23H, methyl- and methylene- of triterpenoid structure). ^13^C-NMR (CDCl_3_) (ppm): 15.4, 15.6, 17.0, 18.3, 21.1, 23.2, 23.5, 24.3, 27.3, 28.0, 28.1, 30.7, 33.0, 36.7, 37.0, 38.7, 38.8, 38.8, 39.1, 39.5, 42.0, 47.5, 48.3, 52.9, 55.2, 79.0, 125.7, 138.0, 176.9 (-COO-); pyrazine ring: 20.6 (-CH_3_), 21.4 (-CH_3_), 21.7 (-CH_3_), 64.8 (-CH_2_), 145.4, 148.8, 149.3, 151.0. HRMS (ESI) *m*/*z*: 591.45245 [M + H]^+^, calcd. for C_38_H_58_N_2_O_3_ 590.44474.

*3β-Hydroxy-lup-20(29)-ene-28-oic acid-3*,*5*,*6-trimethylpyrazin-2-methyl ester* (**4a**). White powder, m.p.: 184.6–185.4 °C, yield 54.1%. ^1^H-NMR (CDCl_3_) (ppm): 0.78, 0.80, 0.82, 0.96, 0.98, 1.69 (s, each, 3H, 6 × -CH_3_), 2.51 (s, 3H, -CH_3_), 2.53 (s, 3H, -CH_3_), 2.57 (s, 3H, -CH_3_), 3.02 (m, 1H), 3.19 (m, 1H), 4.61, 4.74 (each, brs, 1H, =CH_2_), 5.20, 5.23 (each, d, *J* = 12.5 Hz, 1H, -CH_2_), 1.00–2.50 (25H, methyl- and methylene- of triterpenoid structure). ^13^C-NMR (CDCl_3_) (ppm): 14.7, 15.4, 15.9, 16.1, 18.3, 19.4, 20.9, 25.5, 27.4, 28.0, 29.7, 30.6, 32.1, 34.4, 36.9, 37.2, 38.1, 38.7, 38.9, 40.7, 42.4, 46.9, 49.5, 50.6, 55.4, 56.7, 79.0, 109.6, 150.5, 175.5 (-COO-); pyrazine ring: 20.4 (-CH_3_), 21.4 (-CH_3_), 21.6 (-CH_3_), 64.3 (-CH_2_), 145.4, 148.7, 148.9, 150.9. HRMS (ESI) *m*/*z*: 591.45212 [M + H]^+^, calcd. for C_38_H_58_N_2_O_3_ 590.44474.

#### 3.1.2. General Procedure for the Preparation of Ligustrazine-Triterpenes Derivatives **1b**–**4b**

To a solution of ligustrazine-triterpenes derivatives **1a**–**4a** (1.0 mmoL) in dry dichloromethane (25 mL), compound **2** (1.2 mmoL), EDCI (1.5 mmoL), and DMAP (0.1 mmoL) were added. The mixture was allowed to stir at room temperature for 12 h under nitrogen atmosphere. Then the solution was washed with brine. After drying the organic layer over anhydrous Na_2_SO_4_ and evaporating the solvent under vacuum, the crude product was purified by flash chromatography (petroleum ether:acetone = 7:1).

*3β-Hydroxyolea*-*(3,5,6-trimethylpyrazin-2-carbonyl)-12-en-28-oic acid-3*,*5*,*6-trimethylpyrazin-2-methyl ester* (**1b**). White powder, m.p.: 193.0–193.7 °C, yield 53.7%. ^1^H-NMR (CDCl_3_) (ppm): 0.59, 0.92, 0.93, 0.97, 1.15 (s, each, 3H, 5 × -CH_3_), 0.98 (brs, 6H, 2 × -CH_3_), 2.52 (s, 3H, -CH_3_), 2.53 (s, 3H, -CH_3_), 2.58 (brs, 9H, 3 × -CH_3_), 2.73 (s, 3H, -CH_3_), 2.89 (m, 1H), 4.87 (m, 1H), 5.18, 5.24, (each, d, *J* = 12.5 Hz, 1H, -CH_2_), 5.27 (brs, 1H, =CH-), 1.00–2.50 (22H, methyl- and methylene- of triterpenoid structure). ^13^C-NMR (CDCl_3_) (ppm): 15.4, 16.8, 17.0, 18.2, 23.1, 23.4, 23.7, 25.8, 27.6, 28.2, 30.7, 32.4, 32.7, 33.1, 33.9, 37.0, 38.0, 38.2, 39.3, 41.4, 41.7, 45.9, 46.9, 47.5, 55.4, 82.9, 122.4, 143.7, 177.2 (-COO-); pyrazine ring: 20.5 (-CH_3_), 21.4 (-CH_3_), 21.6 (-CH_3_), 22.1 (-CH_3_), 22.7 (-CH_3_), 64.9 (-CH_2_), 140.8, 145.5, 148.8, 149.1, 149.3, 149.9, 150.9, 153.8, 166.2 (-COO-), HRMS (ESI) *m*/*z*: 761.49756 [M + Na]^+^, calcd. for C_46_H_66_N_4_O_4_ 738.50841.

*3β-Hydroxyurs*-*(3,5,6-trimethylpyrazin-2-carbonyl)-12-en-28-oic acid-3*,*5*,*6-trimethylpyrazin-2-methyl ester* (**2b**). White solid, m.p.: 168.2–168.9 °C, yield 46.8%. ^1^H-NMR (CDCl_3_) (ppm): 0.62, 0.98, 1.09 (s, each, 3H, 3 × -CH_3_), 0.87 (d, *J* = 6.0 Hz, 3H, -CH_3_), 0.96 (d, *J* = 6.0 Hz, 3H, -CH_3_), 0.99 (brs, 6H, 2 × -CH_3_), 2.26 (d, *J* = 11.5 Hz, 1H), 2.52 (s, 3H, -CH_3_), 2.54 (s, 3H, -CH_3_), 2.57 (brs, 9H, -CH_3_), 2.73 (s, 3H, -CH_3_), 4.87 (m, 1H), 5.06 (d, each, *J* = 12.0 Hz, 1H, -CH_2_), 5.21 (brs, 1H, =CH-), 5.25, 1.00–2.50 (22H, methyl- and methylene- of triterpenoid structure). ^13^C-NMR (CDCl_3_) (ppm): 15.5, 17.0, 17.0, 17.1, 18.2, 21.2, 23.2, 23.5, 23.7, 24.2, 27.9, 28.2, 30.7, 33.0, 36.7, 36.9, 38.0, 38.4, 38.8, 39.1, 39.6, 42.0, 47.5, 48.3, 52.9, 55.4, 83.0, 125.6, 138.1, 177.0 (-COO-); pyrazine ring: 20.5 (-CH_3_), 21.4 (-CH_3_), 21.6 (-CH_3_), 22.1 (-CH_3_), 22.7 (-CH_3_), 64.8 (-CH_2_), 140.8, 145.5, 148.9, 149.2, 149.3, 149.9, 150.9, 153.8, 166.2 (-COO-). HRMS (ESI) *m*/*z*: 739.51645 [M + H]^+^, calcd. for C_46_H_66_N_4_O_4_ 738.50841.

*3β-Hydroxy*-*(3,5,6-trimethylpyrazin-2-carbonyl)-11-oxoolean-12-en-30-oic acid-3*,*5*,*6-trimethylpyrazin-2-methyl ester* (**3b**). White powder, m.p.: 295.9–296.7 °C, yield 51.7%. ^1^H-NMR (CDCl_3_) (ppm): 0.82, 1.00, 1.01, 1.15, 1.39 (s, each, 3H, 5 × -CH_3_), 1.22 (brs, 6H, 2 × -CH_3_), 2.53 (s, 3H, -CH_3_), 2.54 (s, 3H, -CH_3_), 2.56 (s, 3H, -CH_3_), 2.58 (brs, 6H, 2 × -CH_3_), 2.73 (s, 3H, -CH_3_), 4.89 (m, 1H), 5.20, 5.28 (d, each, *J* = 15.0 Hz, 1H, -CH_2_), 5.58 (s, 1H, =CH-), 1.00–3.00 (21H, methyl- and methylene- of triterpenoid structure). ^13^C-NMR (CDCl_3_) (ppm): 16.4, 17.0, 17.4, 18.7, 23.4, 23.7, 26.5, 26.5, 28.2, 28.4, 28.5, 31.2, 31.9, 32.7, 37.0, 37.7, 38.4, 38.9, 41.1, 43.2, 44.2, 45.4, 48.1, 55.1, 61.7, 82.6, 128.5, 169.0, 199.8 (-C=O), 176.1 (-COO-); pyrazine ring: 20.5 (-CH_3_), 21.4 (-CH_3_), 21.6 (-CH_3_), 22.1 (-CH_3_), 22.6 (-CH_3_), 64.7 (-CH_2_), 140.8, 145.0, 148.4, 149.2, 150.0, 149.3, 151.1, 153.8, 166.2 (-COO-). HRMS (ESI) *m*/*z*: 775.47629 [M + Na]^+^, calcd. for C_46_H_64_N_4_O_5_ 752.48767.

*3β-Hydroxy*-*(3,5,6-trimethylpyrazin-2-carbonyl)-lup-20(29)-ene-28-oic acid-3*,*5*,*6-trimethylpyrazin-2-methyl ester* (**4b**). White powder, m.p.: 142.1–142.9 °C, yield 47.9%. ^1^H-NMR (CDCl_3_) (ppm): 0.82, 0.89, 0.98, 1.70 (s, each, 3H, 4 × -CH_3_), 0.96 (brs, 6H, 2 × -CH_3_), 2.51 (s, 3H, -CH_3_), 2.53 (s, 3H, -CH_3_), 2.57 (brs, 9H, 3 × -CH_3_), 2.72 (s, 3H, -CH_3_), 3.02 (m, 1H), 4.62, 4.74 (each, brs, 1H, =CH_2_), 4.84 (m, 1H), 5.23, 5.20 (d, each, *J* = 12.5 Hz, 1H, -CH_2_), 1.00–2.50 (24H, methyl- and methylene- of triterpenoid structure). ^13^C-NMR (CDCl_3_) (ppm): 14.7, 15.9, 16.2, 16.8, 18.2, 19.3, 20.9, 23.8, 25.5, 28.1, 29.6, 30.6, 32.1, 34.3, 36.9, 37.2, 38.1, 38.5, 40.7, 42.4, 46.9, 49.5, 50.5, 55.5, 56.7, 83.0, 109.7, 150.5, 175.4 (-COO-); pyrazine ring: 20.5 (-CH_3_), 21.4 (-CH_3_), 21.6 (-CH_3_), 21.6 (-CH_3_), 22.1 (-CH_3_), 22.6 (-CH_3_), 64.3 (-CH_2_), 140.9, 145.4, 148.7, 148.9, 149.3, 149.9, 150.9, 153.7, 166.2 (-COO-). HRMS (ESI) *m*/*z*: 739.51637 [M + H]^+^, calcd. for C_46_H_66_N_4_O_4_ 738.50841.

#### 3.1.3. General Procedure for the Preparation of the Intermediates **4**, **5**, **6**, and **7**

The corresponding pentacyclic triterpenes (5.0 mmoL) and benzyl bromide (5.0 mmoL) were dissolved in 25 mL dry DMF, then K_2_CO_3_ (5.0 mmoL) was added and the mixture was kept at 85 °C for 2 h under nitrogen atmosphere. The warm reaction mixture was poured into ice-water and the crude product precipitated out. Then the crude product was filtered and the filtrate was washed with water, dried. The crude product was used without any further purification.

*3β-Hydroxyolea-12-en-28-oic acid-benzyl ester* (**4**). White amorphous solid, m.p.: 188.8–189.5 °C, yield 95.8%. ^1^H-NMR (CDCl_3_) (ppm): 0.63, 0.80, 0.90, 0.92, 0.94, 1.00, 1.15 (s, each, 3H, 7 × -CH_3_), 2.92 (m, 1H), 3.23 (m, 1H), 5.06, 5.12, (d, *J* = 12.4 Hz, 1H, -CH_2_), 5.31 (t, *J* = 3.6 Hz, 1H, =CH-), 7.36 (m, 5H, Ar-H), 1.00–2.50 (23H, methyl- and methylene- of triterpenoid structure).

*3β-Hydroxyurs-12-en-28-oic acid-benzyl ester* (**5**). White powder, m.p.: 76.8–77.4 °C, yield 92.7%. ^1^H-NMR (CDCl_3_) (ppm): 0.67, 0.80, 0.87 (d, *J* = 6.0 Hz, 3H, 3 × -CH_3_), 0.92, 0.96 (d, *J* = 6.0 Hz, 3H, 2 × -CH_3_), 1.01, 1.10 (s, 3H, 2 × -CH_3_), 2.28 (d, *J* = 11.2 Hz, 1H), 3.23 (m, 1H), 5.01, 5.13 (d, each, *J* = 12.4 Hz, 1H, -CH_2_), 5.26 (t, *J* = 3.6 Hz, 1H, =CH-), 7.36 (m, 5H, Ar-H), 1.00–2.50 (24H, methyl- and methylene- of triterpenoid structure).

*3β-Hydroxy-11-oxoolean-12-en-30-oic acid-benzyl ester* (**6**). White powder, m.p.: 92.9–93.7 °C, yield 94.1%. ^1^H-NMR (CDCl_3_) (ppm): 0.76, 0.83, 1.02, 1.13, 1.16, 1.18, 1.37 (s, each, 3H, 7 × -CH_3_), 3.24 (m, 1H), 5.11, 5.22 (d, each, *J* = 12.4 Hz, 1H, -CH_2_), 5.58 (s, 1H, =CH-), 7.39 (m, 5H, Ar-H), 1.00–3.00 (22H, methyl- and methylene- of triterpenoid structure).

*3β-Hydroxy-lup-20(29)-ene-28-oic acid-benzyl ester* (**7**). White powder, m.p.: 191.3–192.1 °C, yield 92.7%. ^1^H-NMR (CDCl_3_) (ppm): 0.78 (brs, 6H, 2 × -CH_3_), 0.82, 0.97, 0.98, 1.70 (s, each, 3H, 4 × -CH_3_), 3.05 (brs, 1H), 3.19 (m, 1H), 4.62, 4.75 (brs, 1H, =CH_2_), 5.11, 5.17 (d, each, *J* = 12.4 Hz, 1H, -CH_2_), 7.38 (m, 5H, Ar-H), 1.00–2.50 (23H, methyl- and methylene- of triterpenoid structure).

#### 3.1.4. General Procedure for the Preparation of Ligustrazine-triterpenes Derivatives **1c**–**4c**

The corresponding intermediates **4**, **5**, **6** and **7** (3.0 mmoL) was dissolved in 25 mL dry DCM, EDCI (3.5 mmoL), DMAP (0.3 mmoL) and compound **2** (3.5mmoL) were added. The mixture was stirred at room temperature for 12 h under nitrogen atmosphere. Then the solution was washed with brine, dried over sodium sulfate, filtered and the solvent was evaporated. Purification was performed by flash chromatography (petroleum ether:acetone = 8:1).

*3β-Hydroxyolea*-*(3,5,6-trimethylpyrazin-2-carbonyl)-12-en-28-oic acid-benzyl ester* (**1c**). White powder, m.p.: 194.2–194.9 °C, yield 47.9%. ^1^H-NMR (CDCl_3_) (ppm): 0.85, 0.92, 0.95, 0.97, 0.98, 0.98, 1.17 (s, each, 3H, 7 × -CH_3_), 2.58 (brs, 6H, 2 × -CH_3_), 2.74 (s, 3H, -CH_3_), 2.89 (m, 1H), 4.87 (m, 1H), 5.07, 5.12 (d, each, *J* = 13.0 Hz, 1H, -CH_2_), 5.32 (brs, 1H, =CH-), 7.35 (m, 5H, Ar-H), 1.00–2.50 (22H, methyl- and methylene- of triterpenoid structure). ^13^C-NMR (CDCl_3_) (ppm): 15.4, 16.9, 17.0, 18.3, 23.1, 23.4, 23.7, 25.9, 27.6, 28.2, 30.7, 32.4, 32.7, 33.1, 33.9, 37.0, 38.0, 38.2, 39.4, 41.4, 41.7, 45.9, 46.8, 47.6, 55.4, 83.0, 122.4, 127.9, 128.0, 128.4, 136.5, 143.8, 177.4 (-COO-); pyrazine ring: 21.6 (-CH_3_), 22.0 (-CH_3_), 22.6 (-CH_3_), 65.9 (-CH_2_), 140.9, 149.4, 149.9, 153.7, 166.2 (-COO-). HRMS (ESI) *m*/*z*: 717.46027 [M + Na]^+^, calcd. for C_45_H_62_N_2_O_4_ 694.47096.

*3β-Hydroxyurs*-*(3,5,6-trimethylpyrazin-2-carbonyl)-12-en-28-oic acid-benzyl ester* (**2c**). White solid, m.p.: 218.5–219.3 °C, yield 51.1%. ^1^H-NMR (CDCl_3_) (ppm): 0.89 (d, *J* = 6.5 Hz, 3H, -CH_3_), 0.98 (brs, 12H, 4 × -CH_3_), 0.68, 1.12 (s, each, 3H, 2 × -CH_3_), 2.29 (d, *J* = 11.5 Hz, 1H), 2.58 (brs, 6H, 2 × -CH_3_), 2.74 (s, 3H, -CH_3_), 4.87 (m, 1H), 5.10, 5.13 (d, each, *J* = 12.5 Hz, 1H, -CH_2_), 5.27 (brs, 1H, =CH-), 7.35 (m, 5H, Ar-H), 1.00–2.50 (22H, methyl- and methylene- of triterpenoid structure). ^13^C-NMR (CDCl_3_) (ppm): 15.5, 17.0, 17.1, 18.2, 21.2, 23.3, 23.6, 23.7, 24.3, 28.0, 28.2, 30.7, 33.0, 36.7, 36.9, 38.0, 38.4, 38.9, 39.1, 39.6, 42.1, 47.5, 48.1, 52.9, 55.4, 83.0, 125.6, 127.9, 128.2, 128.4, 136.4, 138.2, 177.3 (-COO-); pyrazine ring: 21.6 (-CH_3_), 22.0 (-CH_3_), 22.6 (-CH_3_), 66.0 (-CH_2_), 140.9, 149.4, 149.9, 153.7, 166.2 (-COO-). HRMS (ESI) *m*/*z*: 717.46082 [M + Na]^+^, calcd. for C_45_H_62_N_2_O_4_ 694.47096.

*3β-Hydroxy*-*(3,5,6-trimethylpyrazin-2-carbonyl)-11-oxoolean-12-en-30-oic acid-benzyl ester* (**3c**). White powder, m.p.: 212.3–213.0 °C, yield 49.2%. ^1^H-NMR (CDCl_3_) (ppm): 0.76, 1.00, 1.01, 1.14, 1.19, 1.22, 1.39 (s, each, 3H, 7 × -CH_3_), 2.58 (brs, 6H, 2 × -CH_3_), 2.74 (s, 3H, -CH_3_), 4.89 (m, 1H), 5.11, 5.23 (d, each, *J* = 10.0 Hz, 1H, -CH_2_), 5.58 (s, 1H, =CH-), 7.38 (m, 5H, Ar-H), 1.00–3.00 (21H, methyl- and methylene- of triterpenoid structure). ^13^C-NMR (CDCl_3_) (ppm): 16.4, 17.0, 17.4, 18.7, 23.3, 23.7, 26.4, 26.5, 28.2, 28.3, 28.4, 31.2, 31.8, 32.7, 37.0, 37.7, 38.4, 38.9, 41.1, 43.2, 44.0, 45.4, 48.3, 55.1, 61.7, 82.6, 128.2, 128.3, 128.5, 128.6, 136.2, 169.1, 176.2 (-COO-), 199.9 (-C=O); pyrazine ring: 21.6 (-CH_3_), 22.0 (-CH_3_), 22.6 (-CH_3_), 66.2 (-CH_2_), 140.8, 149.3, 149.9, 153.7, 166.2 (-COO-). HRMS (ESI) *m*/*z*: 731.44060 [M + Na]^+^, calcd. for C_45_H_60_N_2_O_5_ 708.45022.

*3β-Hydroxy*-*(3,5,6-trimethylpyrazin-2-carbonyl)-lup-20(29)-ene-28-oic acid-benzyl ester* (**4c**). White powder, m.p.: 162.1–162.8 °C, yield 49.0%. ^1^H-NMR (CDCl_3_) (ppm): 0.80, 0.89, 0.98, 1.71 (s, each, 3H, 4 × -CH_3_), 0.96 (brs, 6H, 2 × -CH_3_), 2.58 (brs, 6H, 2 × -CH_3_), 2.73 (s, 3H, -CH_3_), 3.04 (m, 1H), 4.63 (brs, 1H, =CH_2_), 4.75 (brs, 1H), 4.84 (m, 1H), 5.12, 5.17 (d, each, *J* = 12.5 Hz, 1H, -CH_2_), 7.38 (m, 5H, Ar-H), 1.00–2.50 (24H, methyl- and methylene- of triterpenoid structure). ^13^C-NMR (CDCl_3_) (ppm): 14.7, 15.9, 16.2, 16.8, 18.2, 19.4, 21.0, 23.8, 25.5, 28.1, 29.6, 30.6, 32.1, 34.3, 36.9, 37.2, 38.1, 38.2, 38.5, 40.7, 42.4, 47.0, 49.5, 50.5, 55.5, 56.7, 83.0, 109.6, 128.1, 128.3, 128.5, 136.5, 150.5, 175.8 (-COO-); pyrazine ring: 21.6 (-CH_3_), 22.0 (-CH_3_), 22.6 (-CH_3_), 65.7 (-CH_2_), 140.9, 149.3, 149.9, 153.7, 166.2 (-COO-). HRMS (ESI) *m*/*z*: 717.46016 [M + Na]^+^, calcd. for C_45_H_62_N_2_O_4_ 694.47096.

#### 3.1.5. General Procedure for the Preparation of Ligustrazine-Triterpenes Derivatives **1d**–**4d**

Pd(OH)_2_/C (10%; 80 mg) was added to a solution of compound **1c**–**4c** (1mmoL) in 30 mL MeOH. The mixture was stirred at room temperature for 12 h and filtered to remove Pd(OH)_2_/C. The filtrate was concentrated in vacuum and the residue was purified by flash chromatography (petroleum ether:acetone = 5:1).

*3β-Hydroxyolea*-*(3,5,6-trimethylpyrazin-2-carbonyl)-12-en-28-oic acid* (**1d**). White powder, m.p.: 289.7–290.5 °C, yield 92.8%. ^1^H-NMR (CDCl_3_) (ppm): 0.80, 0.94, 0.96, 0.98, 0.99, 1.00, 1.18 (s, each, 3H, 7 × -CH_3_), 2.57 (brs, 6H, 2 × -CH_3_), 2.73 (s, 3H, -CH_3_), 2.86 (m, 1H), 4.87 (m, 1H), 5.31 (brs, 1H, =CH-), 1.00–2.50 (23H, methyl- and methylene- of triterpenoid structure). ^13^C-NMR (CDCl_3_) (ppm): 15.4, 17.0, 17.2, 18.2, 22.9, 23.4, 23.6, 23.7, 25.9, 27.7, 28.2, 30.7, 32.5, 32.6, 33.1, 33.8, 37.0, 38.0, 38.2, 39.3, 41.0, 41.6, 45.9, 46.5, 47.6, 55.4, 83.0, 122.5, 143.7, 183.4 (-COOH); pyrazine ring: 21.6 (-CH_3_), 22.0 (-CH_3_), 22.6 (-CH_3_), 140.8, 149.3, 149.9, 153.8, 166.2 (-COO-). HRMS (ESI) *m*/*z*: 627.41275 [M + Na]^+^, calcd. for C_38_H_56_N_2_O_4_ 604.42401.

*3β-Hydroxyurs*-*(3,5,6-trimethylpyrazin-2-carbonyl)-12-en-28-oic acid* (**2d**) White solid, m.p.: 305.9–306.6 °C, yield 90.6%. ^1^H-NMR (CDCl_3_) (ppm): 0.81, 1.02, 1.12 (s, each, 3H, 3 × -CH_3_), 0.89 (d, *J* = 6.5 Hz, 3H, -CH_3_), 0.98 (brs, 9H, 3 × -CH_3_), 2.22 (d, *J* = 11.0 Hz, 1H), 2.58 (brs, 6H, 2 × -CH_3_), 2.73 (s, 3H, -CH_3_), 4.87 (m, 1H), 5.27 (brs, 1H, =CH-), 1.00–2.50 (23H, methyl- and methylene- of triterpenoid structure). ^13^C-NMR (CDCl_3_) (ppm): 15.6, 17.0, 17.1, 18.2, 21.2, 23.3, 23.6, 23.7, 24.1, 28.0, 28.2, 30.6, 32.9, 36.7, 37.0, 38.0, 38.4, 38.8, 39.0, 39.6, 42.0, 47.5, 48.0, 52.6, 55.4, 83.0, 125.7, 138.0, 183.4 (-COOH); pyrazine ring: 21.6 (-CH_3_), 22.0 (-CH_3_), 22.6 (-CH_3_), 140.9, 149.4, 149.9, 153.7, 166.2 (-COO-). HRMS (ESI) *m*/*z*: 627.41319 [M + Na]^+^, calcd. for C_38_H_56_N_2_O_4_ 604.42401.

*3β-Hydroxy*-*(3,5,6-trimethylpyrazin-2-carbonyl)-11-oxoolean-12-en-30-oic acid* (**3d**). White powder, m.p.: 305.4–306.2 °C, yield 90.1%. ^1^H-NMR (CDCl_3_) (ppm): 0.87, 1.00, 1.01, 1.17, 1.23, 1.26, 1.42 (s, each, 3H, 7 × -CH_3_), 2.58 (brs, 6H, 2 × -CH_3_), 2.74 (s, 3H, -CH_3_), 4.90 (m, 1H), 5.75 (s, 1H, =CH-), 1.00–3.00 (22H, methyl- and methylene- of triterpenoid structure). ^13^C-NMR (CDCl_3_) (ppm): 16.4, 17.0, 17.4, 18.7, 23.3, 23.7, 26.5, 26.5, 28.2, 28.4, 28.6, 31.0, 31.9, 32.7, 37.0, 37.7, 38.4, 38.9, 40.9, 43.3, 43.8, 45.5, 48.3, 55.1, 61.7, 82.7, 128.5, 169.4, 181.3 (-COOH), 200.2 (-C=O); pyrazine ring: 21.5 (-CH_3_), 22.0 (-CH_3_), 22.5 (-CH_3_), 140.9, 149.4, 149.9, 153.7, 166.1 (-COO-). HRMS (ESI) *m*/*z*: 617.39564 [M − H]^−^, calcd. for C_38_H_54_N_2_O_5_ 618.40327.

*3β-Hydroxy-(3,5,6-trimethylpyrazin-2-carbonyl)-lup-20(29)-ene-28-oic acid* (**4d**). White powder, m.p.: 279.5–280.3 °C, yield 89.2%. ^1^H-NMR (CDCl_3_) (ppm): 0.91, 0.96, 0.97, 0.98, 1.02, 1.72 (s, each, 3H, 6 × -CH_3_), 2.57 (brs, 6H, 2 × -CH_3_), 2.72 (s, 3H, -CH_3_), 3.04 (m, 1H), 4.64, 4.77 (each, brs, 1H, =CH_2_), 4.85 (m, 1H), 1.00–2.50 (25H, methyl- and methylene- of triterpenoid structure). ^13^C-NMR (CDCl_3_) (ppm): 14.7, 16.1, 16.2, 16.8, 18.2, 19.4, 20.9, 23.8, 25.5, 28.1, 29.7, 30.6, 32.2, 34.3, 37.1, 37.2, 38.2, 38.4, 38.5, 40.8, 42.5, 47.0, 49.3, 50.5, 55.5, 56.4, 83.0, 109.7, 150.4, 181.4 (-COOH); pyrazine ring: 21.6 (-CH_3_), 22.0 (-CH_3_), 22.6 (-CH_3_), 140.9, 149.3, 149.9, 153.7, 166.2 (-COO-). HRMS (ESI) *m*/*z*: 627.41288 [M + Na]^+^, calcd. for C_38_H_56_N_2_O_4_ 604.42401.

### 3.2. Bio-Evaluation Methods

#### 3.2.1. Cell Culture

The cell lines Bel-7402 (human hepatocellular carcinoma), HepG2 (human hepatocellular carcinoma), HT-29 (human colon carcinoma), Hela (human cervical cancer), MCF-7 (human breast cancer) were included in this study. All these cell lines were provided by the Chinese Academy of Medical Sciences & Peking Union Medical College. Cultures were maintained as monolayers in RPMI 1640 supplemented with 10% (*v*/*v*) heat inactivated fetal bovine serum and 1% (*v*/*v*) enicillin/streptomycin (Thermo Technologies, New York, NY, USA) at 37 °C in a humidified atmosphere with 5% CO_2_. The ligustrazine derivatives under study were dissolved in DMSO (Sigma, St. Louis, MO, USA) and added at required concentrations to the cell culture. Cells incubated without the preparations served as the control.

#### 3.2.2. Cytotoxicity Assay

The cytotoxicity of the compounds was evaluated on five cancer cell lines (Bel-7402, HepG2, HT-29, Hela, MCF-7) by the standard MTT assay. In short, exponentially growing cells were seeded into 96-well plates at a density 10^4^ cells/mL. The plates were incubated at 37 °C in a humidified 5% CO_2_ atmosphere. Cells were allowed to adhere to the surface for 24 h and then treated with serial dilutions of the compounds for 72 h. Later, aliquots of [3-(4,5-dimethylthiazol-2-yl)-2,5-diphenyltetrazolium bromide] (MTT) solution (20 μL, 5 mg/mL) were added to each well and the incubation was continued for an additional 4 h. Then the supernatant medium was thrown away. The dark blue formazan crystals formed within the healthy cells were solubilized with DMSO (150 μL) and the absorbance was measured in a plate reader (BIORAD 550 spectrophotometer, Bio-rad Life Science Development Ltd., Beijing, China) at 490 nm. Wells containing no drugs were used as control group. The IC_50_ values were defined as the concentration of compounds that produced a 50% reduction of surviving cells and calculated using Logit-method. Tumor cell growth inhibitory rate was calculated in the following Equation (1):

% inhibition = (1 − Sample group OD/Control group OD) × 100%
(1)

#### 3.2.3. Morphological Observation of HepG2 Cells Using Giemsa Staining

The morphological changes in HepG2 cells were examined by Giemsa staining. HepG2 cells were seeded in 6-well plates. The cell density was adjusted to 4 × 10^3^/well and incubated overnight. After 72 h reaction with certain concentrations of **4a** (2, 4, 8 μM), cells were washed with PBS and fixed with cold methanol for 10 min. Methanol was discarded and cells were air-dried. Then fixed cells were stained with 6% Giemsa (Giemsa, Molecular Probes/Invitrogen Life Technologies, Carlsbad, CA, USA) solution for 5 min, washed with water and dried. The cell morphological changes were observed and photographed under a light microscope [38].

#### 3.2.4. Morphological Observation of HepG2 Cells Using DAPI Staining

DAPI staining was used to evaluate apoptosis associated morphological changes in this assay. HepG2 cells were seeded in the six well plates at a density of 4 × 10^3^ cells/well and incubated overnight. Then the cells were incubated in the presence of **4a** at various concentrations (2, 4, 8 μΜ) at 37 °C and 5% CO_2_ for 72 h. Cells were then washed with PBS, fixed with 4% paraformaldehyde followed by 70% ethanol. Finally, fixed cells were stained with DAPI (DAPI, Molecular Probes/Invitrogen Life Technologies, Carlsbad, CA, USA) at the concentration of 1 mg/mL and incubated for 1 min in dark. The cell morphological changes were observed by fluorescent microscopy and images were captured by digital camera [39].

#### 3.2.5. Morphological Observation of HepG2 Cells Using AO/EB Double Staining

Apoptotic study was performed using AO/EB double staining. HepG2 cells were seeded in six well plates at a density of 1 × 10^4^ cells/well and allowed to grow for 24 h. Then the cells were incubated in the presence of **4a** at various concentrations (2, 4, 8 μΜ) at 37 °C and 5% CO_2_ for 72 h. After the treatment period, cells were collected and centrifuged; then the cells were suspended in phosphate-buffer saline (PBS) and centrifuged again. The liquid was removed; the cells were suspended in PBS. Then AO/EB (AO/EB, Sigma, USA) solution (1:1 *v*/*v*) was added to the cell suspension in a final concentration of 100 μg/mL. Cell morphologies were assessed under a fluorescent microscope [29,40].

#### 3.2.6. Apoptosis Analysis by FCM Using Annexin V-FITC/PI Staining

The Annexin V-FITC/PI flow cytometric assay was performed to determine the early and late apoptosis in this assay according to the instruction of the manufacturer (Annexin V-FITC/PI, Beijing BioDee Biotech. Co., Ltd., Beijing, China). Briefly, HepG2 cells were treated with various concentrations of compound **4a** (5, 6, 7 μM) for 72 h at 37 °C, then the treated cells were washed twice with cold PBS and centrifuged at 1000 rpm for 5 min. The harvested cells were resuspended in 200 μL binding buffer, containing 10 μL Annexin V-FITC. After 15 min, the cells were washed twice and resuspended in 300 μL binding buffer. At the same time, 10 μL PI was added. Then the cells were immediately analyzed with a flow cytometry [41].

#### 3.2.7. Mitochondrial Membrane Potential (*ΔΨ*m) Analysis

The mitochondrial membrane potential was measured using rhodamine 123 (Rh123) staining and flow cytometry analysis. HepG2 cells (1 × 10^5^ cells/mL) in logarithmic growth phase were seeded in 6-well culture plate and incuated for 24 h at 37 °C in incubator with 5% CO_2_. After treatment with different concentrations of **4a** (5, 6, 7 μM) for 72 h, cells were collected and washed twice in cold PBS, then resuspended in 1 mL cell culture medium with Rh123 (10 μg/mL) (Rh123, Beijing BioDee Biotech. Co., Ltd., Beijing, China) and incubated for 30 min at 37 °C. After washed twice with cold PBS, the cells were immediately analyzed by the Flow Cytometer at 488 nm excitation wavelength [31,35].

#### 3.2.8. Intracellular Free Ca^2+^ Detection

The level of intracellular free Ca^2+^ was examined by the fluorescent dye Fluo-3AM. HepG2 cells (1 × 10^5^ cells/mL) in logarithmic growth phase were cultured in 6-well plates for 24 h at 37 °C in incubator with 5% CO_2_. After exposed to different concentrations of **4a** (5, 6, 7 μM) for 72 h, cells were harvested and washed twice with cold PBS, then resuspended in HBSS buffer with 10 μM Fluo-3AM (Fluo-3AM, Shanghai Beyotime Biotech. Co., Ltd., Shanghai, China), and incubated for 30 min at 37 °C in the dark. Detection of intracellular Ca^2+^ was carried out by the Flow Cytometer at 488 nm excitation wavelength [35,36].

## 4. Conclusions

In present study, several novel ligustrazine-triterpenes derivatives was designed and synthesized through combination bioactive pentacyclic triterpenes and ligustrazine. Their cytotoxicity was evaluated on five cancer cell lines (Bel-7402, HepG2, HT-29, Hela, and MCF-7) by the standard MTT assay, and their nephrotoxicity was tested using MDCK cells. The ClogP values of those derivatives were calculated by means of computer simulation. The antitumor activities screening indicated that, after coupling with ligustrazine, most of the synthesized compounds showed better antiproliferative activities than the starting materials. It also revealed that most of the derivatives with lower ClogP seemed to exhibit better anti-proliferative effects than those with higher ClogP, as exemplified by **1a**, **1d** > **1b**, **1c**; **2a**, **2d** > **2b**, **2c**; **4a**, **4d** > **4b**, **4c**. In particular, compound **4a** exhibited better cytotoxic activity against Bel-7402, HT-29, MCF-7, Hela and HepG2 than DDP. Meanwhile, most of the candidates exhibited better selectivity towards MDCK cells. For example, at the concentration of 10 μM, the inhibition rate of **4a** against MDCK was just about 26.50%, while the inhibition rate against human hepatoma cell HepG2 was up to 99.87% at the same concentration. Subsequent fluorescence staining and flow cytometry analysis indicated that **4a** could induce apoptosis in HepG2 cell line. Further apoptosis mechanism investigation showed that **4a** could also induce the depolarization of mitochondrial membrane potential and the disturbance of intracellular free Ca^2+^ concentration. Further studies of the mechanisms of these compounds cytotoxic selectivity in human malignant tumors are currently underway. The results suggest that the attempt to apply structure combination to discover more efficient and lower toxicity anti-tumor lead compounds from nature products is viable.
